# Superparamagnetic Iron Oxide for Identifying Sentinel Lymph Node in Breast Cancer after Neoadjuvant Chemotherapy: Feasibility Study

**DOI:** 10.3390/jcm10143149

**Published:** 2021-07-16

**Authors:** Andrzej Kurylcio, Zuzanna Pelc, Magdalena Skórzewska, Karol Rawicz-Pruszyński, Radosław Mlak, Katarzyna Gęca, Katarzyna Sędłak, Piotr Kurylcio, Teresa Małecka-Massalska, Wojciech Polkowski

**Affiliations:** 1 Department of Surgical Oncology, Medical University of Lublin, Radziwiłłowska 13 St., 20-080 Lublin, Poland; andrzej.kurylcio@umlub.pl (A.K.); zuzanna.torun@gmail.com (Z.P.); magdalena.skorzewska@umlub.pl (M.S.); katarzyna.geca@umlub.pl (K.G.); sedlak.katarz@gmail.com (K.S.); p.kurylcio@gmail.com (P.K.); wojciech.polkowski@uml.edu.pl (W.P.); 2 Department of Human Physiology, Medical University of Lublin, Radziwiłłowska 11 St., 20-080 Lublin, Poland; radoslaw.mlak@uml.edu.pl (R.M.); teresa.malecka-massalska@umlub.pl (T.M.-M.)

**Keywords:** breast cancer, neoadjuvant chemotherapy, sentinel lymph node, SPIO

## Abstract

Sentinel lymph node biopsy (SLNB) is a well-established procedure for staging clinically node-negative early breast cancer (BC). Superparamagnetic iron oxide (SPIO) demonstrated efficacy for nodal identification using a magnetic probe after local retroaeroal interstitial injection. Its benefits lie in its flexibility, which is an essential property in the global setting, where access to the isotope is difficult. To the best of our knowledge, this is the first study to evaluate the feasibility and safety of the SPIO for SLNB in BC patients treated with neoadjuvant chemotherapy (NAC). Seventy-four female patients were included. The median time of lymph node retrieval was 20 min. The median number of resected sentinel nodes (SNs) was 4. SN was detected in all patients. No serious adverse event was observed. SPIO in identifying SN in BC patients after NAC is feasible and oncologically safe.

## 1. Introduction

Sentinel lymph node biopsy (SLNB) is a well-established procedure for staging clinically node-negative early breast cancer (BC), although the optimal surgical management of the axilla has been controversially discussed over the last two decades [1]. Due to its minimally invasive approach and low morbidity, SLNB established a new milestone replacing axillary lymph node dissection (ALND) [2,3]. Thus, the quality of life in BC patients undergoing nodal staging has improved significantly, with a substantial reduction of postoperative seroma, numbness, lymphodema, chronic pain or shoulder mobility problems [4,5,6,7,8]. Sentinel node (SN) detection in BC was introduced by Giuliano in 1994, using blue dye [3]. Krag [2] and Veronesi [9] implemented the use of radiotracer, Technetium-99m (Tc99) labeled nano colloid with a handheld gamma probe for SN identification. The simultaneous use of a radioisotope with blue dye increased the SN detection rate up to 96–97%, as shown in the AMAROS and ALMANAC trials [10,11]. The use of a radioactive tracer alone or in combination with blue dye has been recognized as a gold standard of SLNB [4,10].

However, the existing drawbacks of the dual method, such as radiation exposure, nuclear medicine unit dependency, substantial legislative control and allergic reactions to blue dye, warranted a clinical need for new, non-radioactive methods of SN identification. Superparamagnetic iron oxide (SPIO) nanoparticles demonstrated efficacy for nodal using a magnetic probe before local retroaeroal interstitial injection. Clinical studies and meta-analyses compared the magnetic technique with standard radioactive localization in BC [12,13,14,15,16,17,18,19,20,21,22,23] and revealed non-inferiority to the gold standard isotope technique, with or without blue dye. The SPIO particles have been used for more than 20 years as an intravenous contrast for magnetic resonance imaging [24]. SPIO has a comparable detection rate as the dual technique and has not been associated with allergic reactions [25]. It remains in SNs after injection in the breast for more than 30 days [23] and thus provides independence from nuclear medicine units. SPIO benefits lie in its flexibility, which is an essential property in the global setting where access to the isotope is difficult [25]. Magtrace (Endomagnetics Limited, Cambridge, UK) is a dark brown–brownish suspension of a dextran-coated nanoparticle (60 nm) [25]. It is designed for lymphatic uptake and filtering out in the SNs [26]. In previous studies, Sienna+ (Endomagnetics Limited), a magnetic tracer, was used, however, it required dilution with saline before injection. On the contrary, Magtrace, which contains the same SPIO nanoparticles, does not require dilution. Thus the injected tracer volume can be reduced. Magnetic particles are detected using a handheld magnetometer (SentiMag). Neoadjuvant chemotherapy (NAC) in early BC results in higher rates of breast-conserving therapy when compared to adjuvant chemotherapy, without compromising on distant recurrence, breast cancer survival or overall survival [27,28]. NAC is currently the standard of care in locally advanced BC [29]. For early BC management, ESMO recommends a treatment regimen based on anthracyclines (with or without taxanes), which reduces BC mortality by 30% and improves the efficacy of NAC independently of LN involvement [30]. According to the NCCN BC Guidelines, SLNB is recommended in patients with nodes clinically negative after NAC [31]. Proper LN status assessment remains crucial for further decisions considering adjuvant treatment [32]. Moreover, nodal staging after NAC reflects more accurately future prognosis than LN assessment before neoadjuvant treatment [33]. To the best of our knowledge, there are no reports on the usage of SPIO in SLN evaluation in BC after NAC. Therefore, this study aimed to evaluate the feasibility and safety of the SPIO for SLNB in BC patients treated with NAC.

## 2. Materials and Methods

After obtaining institutional review board approval (Bioethical Committee of the Medical University of Lublin, Ethic Code: Ke-0221-34-2013), we collected data from a prospectively maintained database of all patients operated on in early BC between February 2013 and December 2020 in the Department of Surgical Oncology, Medical University of Lublin, Poland. Some 74 patients were eligible for analysis.

### 2.1. Neoadjuvant Chemotherapy

NAC was administered according to national guidelines, depending on the clinical stage of the disease and molecular subtype. NAC preferred regimen was 4 cycles AC (doxorubicin 75 mg/m^2^ with cyclophosphamide 750 mg/m^2^) administered every 3 weeks (conventional) or 2 weeks (dose-dense), followed by 12 cycles of weekly paclitaxel (80 mg/m^2^) or docetaxel at three-week intervals (100 mg/m^2^). Human epidermal growth factor receptor (HER2)–positive patients additionally received HER2-targeted therapy.

### 2.2. Sentinel Lymph Node Biopsy

We used a handheld magnetometer (SentiMag^®^, (Sysmex Europe GmBH, Hamburg, Germany) to detect the SNs, both before and after skin incision. As magnetic tracers we used Sienna+^®^, (Sysmex Europe GmBH, Hamburg, Germany) (2 mL diluted in saline, obtaining a final volume of 5 mL) and Magtrace^®^ (Endomagnetics Limited, Cambridge, UK), (from 06.2019; undiluted 1 mL). SPIO injection may cause skin staining for more than a year. This is seen almost exclusively after BCS. To avoid skin staining, SPIO was injected deeply into the subareolar interstitial tissue at least 18–24 h before SLNB. Before skin incision, the SPIO injection site and the hot spots of the axillary area were measured with the SentiMag^®^ probe. In order to avoid interference with the magnetometer, polymer retractors and forceps were used while detecting the SLNs with the SentiMag^®^ probe. All LNs marked with SPIO tracer were excised. The intraoperative SLNs identification was based on the node’s handheld magnetometer’s indications and/or brown staining. All identified SN were removed until the background signal was less than 10% of its highest value during SLNB. Therefore, SLNB was stopped when the residual activity in the axilla was less than 10%.

### 2.3. Statistical Analysis

All statistical analyses were performed using MedCalc 15.8 (MedCalc Software, Ostend, Belgium). Median, interquartile range and minimum–maximum were used to summarize continuous variables when appropriate, and frequency and percentage were used to summarize categorical variables.

## 3. Results

### 3.1. Clinical and Pathological Characteristics of the Enrolled Patients

The study group consisted of female patients only. The median age was 55.5 years (range: 26–83), and the median BMI was 26 (17.2–42.2). The highest percentage of tumors was located in the upper outer quadrant (43%). The most common diagnosis was invasive ductal carcinoma (71.6%). Nine patients (8.2%) were clinically node-positive (cN+) prior to NAC, whereas all patients were clinically node-negative after neoadjuvant treatment (ycN0) in ultrasonographical re-staging. Over half of the patients had complete tumor regression ypT0 (56.8%), whereas sixty-one patients had pathologically negative LNs, ypN- (82.4%). Most of the patients had a positive ER (63.1%) and PR (60%) receptor status. However, HER2 expression was increased in only 32.4% of the cases. A high level of Ki67 was recorded in 75.4% of patients. Detailed demographic and clinical characteristics of the patients enrolled in the study are shown in Table 1.

### 3.2. Perioperative Characteristics of the Enrolled Patients

Thirty-seven patients (50%) underwent breast-conserving surgery. Surgical margin was achieved in seventy patients (94.6%). The median time of lymph node retrieval was 20 min (range: 10–50). Lymphadenectomy was performed in thirteen patients (17.6%). The median number of resected SNs was 4 (range: 1–10). SN was detected in all patients. No serious adverse event was observed. Detailed perioperative data is shown in Table 2.

## 4. Discussion

**Figure 1:**
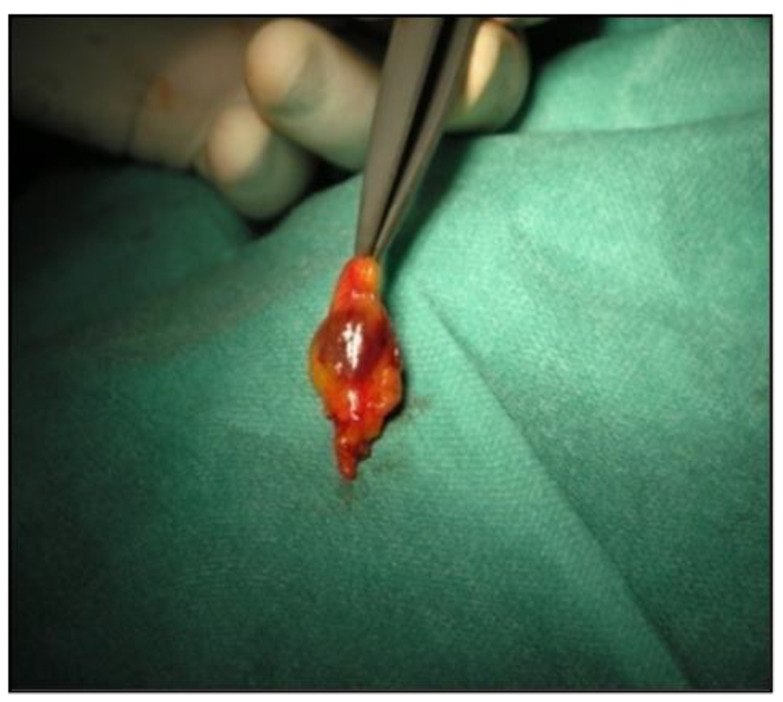
Sentinel node retrieved with superparamagnetic iron oxide (SPIO).

Although Rubio et al. provided a report on the outcome of SLN after NAC using a dual tracer (SPIO-TC99) at ASCO annual meeting [34], to the best of our knowledge, this is the first study that evaluated the feasibility and safety of SPIO for SLNB in BC patients after NAC. SLNs (Figure 1) were successfully identified in all patients, and no postoperative complications were observed. The tumor recurrence after initial response to treatment remains a clinical challenge, despite recent advances in BC management [35]. Moreover, the appropriate axillary treatment in the context of NAC is not clearly established. We have demonstrated that SPIO-guided SLNB could be successfully used in SN detection after NAC (100%). A mean number of resected SLN was four. When harvesting two or three SLN, the false-negative ratio (FNR) remains under 10%, which is considered oncologically safe [36,37,38,39].

SPIO may be a potentially optimal tracer for SLNB after NAC in BC patients due to high SN retrieval number and low FNR compared with conventional methods [40]. Bezire et al. evaluated the feasibility of and the tolerance to radiotherapy after using the magnetic detection method for SLNB. The SN identification rate with SPIO was 99.7%, which is consistent with our result. MONOS study [41] evaluated the use of SPIO as a sole tracer and the efficacy of tracer injection in the preoperative setting. SPIO was concluded as a safe and cost-effective alternative to dual technique using 99m Tc and blue dye with simplified logistics, improved performance and skin staining prevention. When compared to dual-tracers, usage of SPIO saves over 20,000$ annually [23]. Alarcon et al. assessed the efficacy and accuracy of the clipped node’s wire localization to reduce the false-negative rate of SN retrieval. For intraoperative localization, wire marking has proven to be a safe procedure in which removal of SN was achieved in all patients [42]. Similarly, the detection rate of SPIO-guided SLNB in our study was 100%, resulting in 0% FNR. NCCN Guidelines recommend SLNB in selected cases with ycN0 status. Among cN+ patients, SLNB after NAC has a >10% false-negative rate. Removal of at least 3 SN improves this rate. In our study, median SN retrieval was 4. In a study conducted by Wong et al., patients with stage I-III BC who underwent NAC demonstrated acceptable short-term locoregional control associated with SLNB alone in cN1-2/ypN0 patients. SLNB was the most accurate minimally invasive method for staging the axilla and evaluating residual nodal disease [43]. Chun et al., in an analysis of 676 patients, suggested SLNB alone may be a possible option for patients with 1–3 SN+ BC after NAC without compromise of recurrence or overall survival [44]. Since SLN surgery in node-positive patients after NAC is not oncologically inferior to ALND for locoregional recurrence, it is becoming more widely incorporated into clinical practice [45]. With advances in genomics, imaging, and multimodal therapy, the role and extent of axillary surgery for staging should be continually reevaluated [45]. On the other hand, van der Noordaa et al. recently suggested omitting SNLB in patients with triple-negative BC, HER2+ tumors or after achieving breast radiological complete response in MRI [46]. In the EUBREAST-01 trial, axillary surgery will be eliminated for initially clinical node-negative patients with complete radiologic remission and breast pCR in the lumpectomy specimen [47]. Considering the increasing number of patients qualified for NAC, SLNB remains an essential tool for evaluating systemic treatment from the oncological perspective. According to Erdahl et al., evaluation of the N stage after NAC results in a more adequate prediction of the patient’s residual disease severity and response to systemic therapy [32]. These outcomes further determine adjuvant treatment, targeted or hormone therapy for patients previously treated with NAC [48]. However, preoperative therapy increases the complexity of SNLB, indicating the need for tailored, multidisciplinary management based on clinical counseling and standardization of qualification for SNLB after NAC [49]. SIPO-guided SLNB reduces the hospitalization rate and omits lymphoscintigraphy in the nuclear medicine unit, thus possibly shortening the treatment period. This is coherent with the clinical focus on reducing delays between individual treatments in BC patients to improve overall survival [50]. Magnetic-guided SLNB can omit isotope-based axillary mapping and allows a novel tailored approach to BC patients [51].

## 5. Conclusions

SPIO is a feasible and oncologically safe method for identifying SN in BC patients after NAC.

## Figures and Tables

**Table 1:** Demographic and clinical characteristics of the patients.

Variable	Study Group (*n* = 74)
Age (years)	
median	55.5
interquartile range	43–64
min-max	26–83
≥65	18 (24.3%)
<65	56 (75.7%)
BMI (kg/m^2^)	
Median (26)	26
Interquartile range	22.2–28.6
Min–max	17.2–42.2
Underweight (<18.5)	1 (1.4%)
Normal (18.5–24.9)	27 (36.5%)
Overweight (25.0–29.9)	32 (43.2%)
Obese (≥30.0)	14 (18.9%)
Tumor location in the breast	
Central	17 (23%)
Upper outer quadrant	32 (43.1%)
Upper inner quadrant	2 (2.7%)
Lower inner quadrant	10 (13.5%)
Histologic type	
Invasive ductal carcinoma	53 (71.6%)
Non-special type	15 (20.3%)
Other	6 (8.1%)
Nuclear grade	
G1	7 (9.5%)
G2	32 (43.2%)
G3	35 (47.3%)
ypT	
pT0	42 (56.8%)
pT1a	2 (2.7%)
pT1b	1 (1.4%)
pT1c	17 (23%)
pT2	12 (16.2%)
ypN	
Negative	61 (82.4%)
Positive	13 (17.6%)
Molecular subtype	
Luminal A	6 (8.1%)
Luminal B (HER2 negative)	23 (31.2%)
Luminal B (HER2 positive)	16 (21.6%)
HER 2+	7 (9.4%)
Triple negative	22 (29.7%)

BMI—Body Mass Index.

**Table 2:** Perioperative characteristics.

Variable	Study Group (*n* = 74)
Type of breast surgery	
BCS	37 (50%)
MRM	1 (1.4%)
SM	23 (31.1%)
NSM+IBR	13 (17.6%)
Largest dimension of the tumor (mm)	
median	20
interquartile range	13–30
min-max	1.5–60
Surgical margin	
R0	70 (94.6%)
R1	4 (5.4%)
Time of lymph node resection (min)	
median	20
interquartile range	18.7–25
min-max	10–50
SN (number, resected)	
median	4
interquartile range	3–5
min–max	1–10

BCS, breast-conserving surgery; MRM, modified radical mastectomy; SM, simple mastectomy; NSM+IBR, nipple-sparing mastectomy with immediate breast reconstruction; SN, sentinel node.

## Data Availability

Not applicable.
